# Design and Demonstration of a Microelectromechanical System Single-Ring Resonator with Inner Ring-Shaped Spring Supports for Inertial Sensors

**DOI:** 10.3390/s23229234

**Published:** 2023-11-16

**Authors:** Imran Khan, Ahmad Rahbar Ranji, Gnanesh Nagesh, David S.-K. Ting, Mohammed Jalal Ahamed

**Affiliations:** MicroNano Mechatronics Laboratory, Department of Mechanical, Automotive and Materials Engineering, University of Windsor, Windsor, ON N9B 3P4, Canada; khan1cm@uwindsor.ca (I.K.); ahmad.rahbar-ranji@uwindsor.ca (A.R.R.); nagesh@uwindsor.ca (G.N.); dting@uwindsor.ca (D.S.-K.T.)

**Keywords:** finite element analysis, MEMS, ring resonator, mode matching, gyroscope, inertial sensor

## Abstract

This paper presents a novel single-ring resonator design and experimentally demonstrates its dynamic behavior. The proposed ring resonator design is simple and has a solid anchor at its center connected to an outside ring via inner ring-shaped springs. The mode shapes and frequency of the ring resonator were determined numerically and compared with analytical approaches, and the minimum split frequency was observed for the *n* = 3 mode of vibration. Numerical and analytical methods were used to determine the resonance frequencies, pull-in voltage, resonance frequency shift and harmonic response of the ring resonator for different silicon orientations. The split frequency in the *n* = 3 mode of vibration increases by the applied DC bias voltage almost by the same amount for all types of silicon. When an AC voltage with a 180-degree phase is applied to two opposite electrodes, the ring has two resonance frequencies in mode *n* = 2, and when the AC voltage applied to two opposite electrodes is in the same phase, the ring has one resonance frequency regardless of the crystal orientation of silicon. Prototypes were fabricated using a double silicon-on-insulator-based wafer fabrication technique and were tested to verify the resonator performance.

## 1. Introduction

Microelectromechanical system (MEMS) resonators consist of a miniature vibrating mass acting as the sensing element that experiences a Coriolis force under rotation. The Coriolis force acts perpendicular to the direction of the vibrating force and is proportional to both the angular velocity of the rotating object and the linear velocity of the vibration. The Coriolis acceleration transfers energy from the primary mode of vibration (drive mode) to the degenerate mode of vibration (sense mode). This is why the MEMS vibrating gyroscope is also known as a Coriolis vibrating gyroscope (CVG).

Since the detection of rotational velocity is dependent on the transformation of vibration modes, the structure of the vibrating gyroscope plays an important role in producing the desired transformable vibration modes. The vibrating mass is the crucial sensing component, and many vibrating mass shapes have been developed over the years. In summary, vibrating gyroscopes can be classified into various structural shapes, such as cubical, disk, and ring shapes, and their advantages and disadvantages were discussed in [1,2]. Cubical mass structures [3,4,5,6,7], including single mass [3], dual-mass [4], tuning fork structures [5,6], and quadrilateral mass structures [7], have limitations due to the coupling of the driving and sensing axes [3,4,5,6]. Using a higher number of masses can mitigate the coupling effect but may add complexity to the fabrication and measurement of system performance.

Circular structures (disk and hemispherical) [8,9] involving one mass are preferred because of symmetry; however, releasing the disk structure is challenging. The vibrating ring gyroscope (VRG) is a promising type of MEMS gyroscope. The major characteristics that make VRGs unique from other vibratory gyroscopes include balanced, symmetrical structures, equal resonance frequencies in driving and sensing modes, high sensitivity, and low-temperature sensitivity [10,11,12]. The ring structure [10,11,12,13,14,15,16,17,18,19,20,21,22,23,24] with support springs offers ease in structure release compared with cubical and disk structures. It consists of a solid anchor connected to an outer ring via springs of different shapes.

Jia et al. [10] discussed in detail the advantages and disadvantages of vibrating ring gyroscopes. The common key performance factors of a MEMS vibrating ring gyroscope are the driving and sensing resonance frequencies, sensitivity, drift rate, Q factor, and noise error [10,11,12,13,14,15,16,17,18,19,20,21,22,23,24]. The state of the art concerning the above characteristics is discussed in the following paragraphs.

The operation of gyros depends on the driving and sensing mode frequencies. These frequencies can be matched, known as mode-matched, or unmatched, known as a split mode. The mode-matched frequency is desirable for highly sensitive gyros. VRGs are widely investigated because of their intriguing degenerate symmetrical mode shapes and the possibility of applying them as a whole-angle gyroscope without much difficulty. The driving and resonance frequencies in the VRG are highly dependent on the shape and design parameters of the support springs. One research group [12,13,14,15] presented a vibrating ring with eight semicircle spring supports following an initial demonstration [11] using a nickel electroplating method that resulted in a resonance frequency of 33 kHz. Later, a high aspect ratio (width of 4 μm and thickness of 80 μm) capable of producing a degenerate flexural mode frequency at 29.28 kHz with zero split frequency was demonstrated [14]. Yoon et al. [17] used a different design of eight support springs connected with a ring of 3000 μm radius, a thickness of 18 μm, and a height of 150 μm. The operating range resonance frequency was measured to be 17.10 kHz. The value of maximum stress under a shock of 15,000 g was determined to be 200 MPa. Another design of the vibrating ring gyroscope comprises concentric rings and eight C-shaped opposing springs connected to the outer ring, producing a wineglass mode shape at a resonance frequency of 36.67 kHz [22]. Khan et al. [23] introduced a new design consisting of a “rose petal” spring-supported ring with a resonance frequency of 64.89 kHz in the *n* = 2 mode of vibration.

Research shows that various commercial MEMS gyroscopes work well on the principle of split mode frequencies but suffer from noise and a high level of power consumption [24]. Mode-matched frequency is an alternative method to reduce noise and lower power consumption [24]. Research along this line shows that electrical filters and noise compensatory circuits are effective for tuning and matching the frequencies of the driving and sensing modes [25]. Shu et al. [26] introduced another method for mode matching based on geometry compensation.

The ratio of the output amplitude (sense) to input amplitude (drive) is called the angular-gain factor, or the geometric scale factor or sensitivity and is expressed in mV/(deg./s). The input angular velocity (Ω) is calculated by dividing the output quantity by the scale factor [10]. The sensitivity of the VRG can be estimated using a rotating table. Yoon et al. [17] verified that there is an approximately linear relationship between the output voltage and the speed of rotation for a ring resonator from −300 deg./s to +300 deg./s. Ayazi and Najafi [14] achieved 0.2 mV/(deg./s) sensitivity for a ring gyroscope at a bandwidth of 5 Hz. Later, the sensitivity of the same design was developed to 132 mV/(deg./s) using single-crystal silicon [15]. Yoon et al. [16] discussed in detail the sensitivity and mode shape of the same design. Li et al. [18] introduced a ring gyroscope supported with crab-type outer springs that resulted in a sensitivity of 0.1146 μV/(deg./s), and the initial frequency split was only 0.5 Hz. Kou et al. [19,20,21] used an s-type inner support spring in a vibrating ring and produced a 20.2 Hz frequency split between the two operating modes. The sensitivity (scale factor) of the prototype was measured at 4.32 mV/(deg./s) using a rate table [19]. The same design was simulated to calculate the maximum displacement (2.91 μm) and mechanical sensitivity (0.0036 μm/deg.) [21].

The mode-matched gyroscope produced higher sensitivity than the split-mode gyroscope. Gando et al. [27], using the catch-and-release operation technique, improved the gyroscope sensitivity 52 times when reducing the frequency split from 2 kHz to 50 Hz. Higher sensitivity is desirable for sensitive applications such as GPS navigation [2]. The sensitivity of a concentric ring gyroscope can also be improved by varying the uniform and nonuniform width (thickness) of the ring from the outer ring to the inner ring of the gyroscope [28].

Vibrating ring gyroscopes have significant potential usage in the future for high-end inertial applications. The main design differentiator among the various ring resonators is the way the support springs connect the ring to the anchor. In this study, a new spring mechanism for ring-shaped resonators was presented with potential application as a mode-matched gyroscope. The proposed VRG has a simple shape, one solid anchor, and four eccentric rings.

The main objective of this study is to introduce a novel design of a VRG gyroscope compatible with standard silicon-on-insulator (SOI)-based fabrication and demonstrate its applicability. Details of the mechanical design of the ring geometry are provided in the next section, followed by the working principle, mode shapes of the ring resonator and dimensions of the device. Numerical calculations of the working frequencies, pull-in voltage, shift of frequency and harmonic response are given in section three. The fabrication and experimental validation of the proof of concept of the design are discussed in section four, followed by the conclusion.

## 2. Design Features

**Figure 1:**
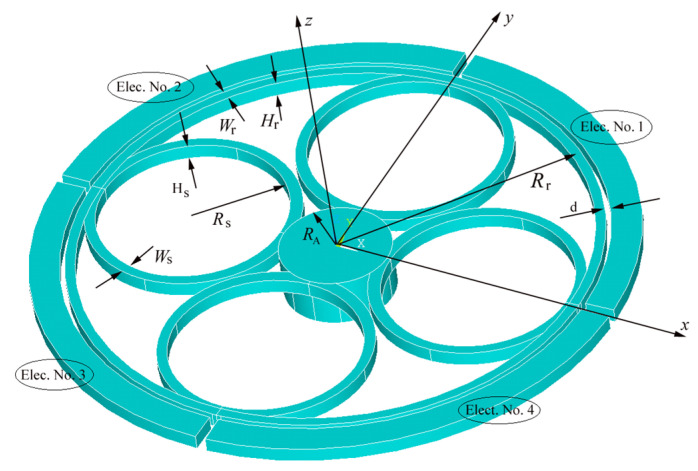
Three-dimensional schematic of the proposed ring resonator and definition of geometrical parameters.

Disk- and ring-shaped resonators are symmetric; however, the large mass of the disk structure makes releasing a challenging task. The ring structure provides more control to tune the stiffness of the spring for the desired mode shape and frequency. Many ring resonators in the form of single- or multiple-rings with different forms of supporting beams inside (spoke) or outside the ring have been proposed. Figure 1 depicts the geometry of the desired ring resonator in this study. It consists of a solid anchor at the center. The ring is supported by four inner ring-shaped springs (spokes). The similarity of the shape of the support springs and outer ring and ease of fabrication make this novel design unique. The design is compatible with fabrication using single silicon on insulator (SOI) wafer wet/dry release-based conventional fabrication.

**Figure 2:**
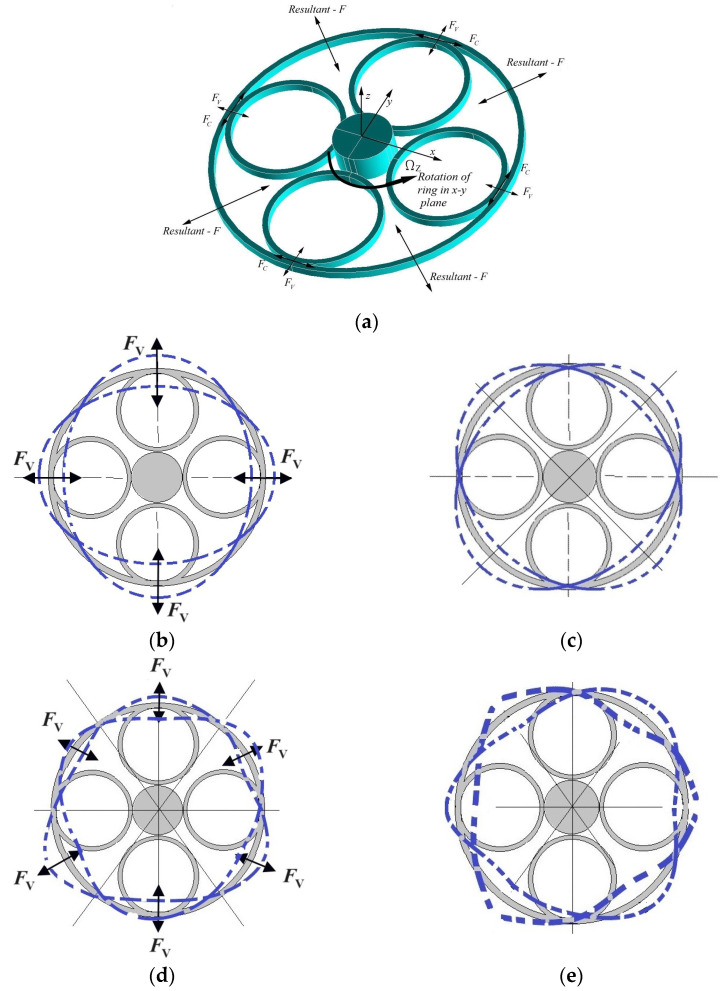
Three-dimensional schematic view showing the resonator with all forces on the ring and *n* = 2 and *n* = 3 mode shapes of vibration; (**a**) The ring resonator with all forces on the ring; (**b**) Driving mode at *n* = 2 mode of vibration; (**c**) Sensing mode at *n* = 2 mode of vibration; (**d**) Driving mode at *n* = 3 mode of vibration; (**e**) Sensing mode at *n* = 3 mode of vibration.

A vibrating ring resonator works on the principle of the Coriolis force. External force is applied to vibrate the ring in flexural mode. While the structure is vibrating, a Coriolis force is generated under the rotation, which is directly proportional to the speed of rotation. A demonstration of the Coriolis force and its effect on the proposed resonator is shown in Figure 2. When the resonator is excited with an electrical voltage applied via electrodes, the electrostatic force, $F_{V}$, between the electrodes and the outer ring is induced (Figure 2b–e). Depending on the arrangement of the electrodes, this force causes the ring to vibrate in *n* = 2 (Figure 2b) or *n* = 3 (Figure 2d) driving mode. If an external rotation is applied, the Coriolis forces, $F_{C}$, are generated perpendicular to the direction of the electrostatic forces (Figure 2a). As seen, in the Figure 2a, there are two Coriolis forces acting in x and y directions that are equal due to the symmetry of the ring. The resultant force of these two acts 45 degrees with respect to the drive axis, inducing the ring to vibrate perpendicular to this axis, called the degenerate mode (sensing mode), close to the wine glass mode shape in *n* = 2 and *n* = 3 mode shapes (Figure 2c–e).

The device operation is intended for *n* = 3 to reduce possible mismatch effects. The *n* = 3 mode of vibration has a higher frequency with a slightly lower displacement, but it ensures minimization of the possible asymmetry effect. The initial values of the considered dimensions of the proposed design ring resonator are given in Table 1. Based on a previous study (Khan et al. [23]), the ratio of the outer ring to the anchor radius is selected as 5.0 to provide enough support to hold the structure and release the moving part of the structure during fabrication.

To drive the ring, four electrodes with a gap distance of 10 μm are designed (Figure 1).

## 3. Numerical Analysis

To investigate the dynamic behavior of the ring resonator with the proposed geometry of the spokes, the computer code ANSYS v. R.2022 [29] has been used, and natural frequencies, pull-in voltage, shift of resonance frequency and harmonic response of the ring are determined. The ring is modeled using SOLID187, a 3D 10-node tetrahedral structural solid with three degrees of freedom at each node. The anchor and the electrodes are fixed in the radial and tangential directions.

### 3.1. Natural Frequency Analysis

#### 3.1.1. Ring Resonator Made of Silicon <111> and Spokes Tangent to the Outside Ring and Anchor

To study the effects of the material properties and geometry of the spokes, first, the material is considered silicon <111>, and the spokes are considered tangent to the anchor and outside ring. The material is considered an isotropic material with a Young’s modulus of E=179 GPa, a Poisson’s ratio of ν=0.28, and a density of ρ=2329 kgm3. The natural frequencies are 201.59 kHZ and 55.27 kHz in *n* = 2 and 223.82 kHz and 223.82 kHz in *n* = 3 for drive and sense modes, respectively.

Rahbar Ranji et al. [30] provided the following equations for the calculation of the natural frequency of the *n*th mode of vibration for drive and sense modes:

(1) $${\left(\omega_{n-Drive}\right)}^{2}=\frac{\frac{{\left(1-n^{2}\right)}^{2}}{{\left(R_{r}\right)}^{3}}\;\pi EI_{r}+k_{sr}\;\sum_{i=1}^{N_{s}}{\left(\cos n\theta_{i}\right)}^{2}+\frac{1}{n^{2}}\;k_{st}\sum_{i=1}^{N_{s}}{\left(\sin n\theta_{i}\right)}^{2}}{\frac{1+n^{2}}{2n^{2}}\;M_{r}+m_{sr}\;\sum_{i=1}^{N_{s}}{\left(\cos n\theta_{i}\right)}^{2}+\frac{1}{n^{2}}m_{st}\sum_{i=1}^{N_{s}}{\left(\sin n\theta_{i}\right)}^{2}}\;{\left(\omega_{n-Sense}\right)}^{2}=\frac{\frac{{\left(1-n^{2}\right)}^{2}}{{\left(R_{r}\right)}^{3}}\pi EI_{r}+k_{sr}\;\sum_{i=1}^{N_{s}}{\left(\sin n\theta_{i}\right)}^{2}+\frac{1}{n^{2}}\;k_{st}\sum_{i=1}^{N_{s}}{\left(\cos n\theta_{i}\right)}^{2}}{\frac{1+n^{2}}{2n^{2}}\;M_{r}+m_{sr}\;\sum_{i=1}^{N_{s}}{\left(\sin n\theta_{i}\right)}^{2}+\frac{1}{n^{2}}m_{st}\sum_{i=1}^{N_{s}}{\left(\cos n\theta_{i}\right)}^{2}}$$

where *E* is the Young modulus, $I_{r}$ is the moment of inertia of the ring with respect to the rotation axis, $R_{r}$ is the mean radius of the ring, $k_{sr}$ and $k_{st}$ are the stiffness of one spoke in the radial and tangential directions of the outside ring, respectively, $m_{sr}$ and $m_{st}$ are the effective masses of one spoke when vibrating in the radial and tangential directions, respectively, $\theta_{i}$ is the position angle of the ith spoke in respect to *x* axes in degree, and $M_{r}$ is the total mass of the ring.

Calculation of the stiffness of spokes is crucial for the determination of natural frequency. For folded beams with any number of beams and the orientation of the spoke when fixed at one end and free to slide at the other end in any direction, Rahbar Ranji et al. [31] gave equations for the calculation of stiffness. For curved beams fixed at one end free to slide at the other end, Rahbar Ranji et al. [30] gave the following equation for the calculation of stiffness:

EIsrs3ks=β+12sin2βcos2α−2βcos2αsin2β−12sin2β−1βsin2β2β−12sin2βcos2α−2βsin2αsin2β sin22α   

where 2β is the central angle of the spoke, α is the direction in which the free end can slide, E is Young’s modulus, $I_{s}$ is the moment inertia with respect to the axis of bending, and $r_{s}$ is the radius of the spoke. To calculate $k_{sr}$ and $k_{st}$, α is considered zero and 90 degrees, respectively. To calculate the effective mass of the spoke in the radial and tangential directions, FEM is used, and the natural frequencies of the curved beam are determined. Having natural frequencies and stiffness, the effective mass is calculated. Table 2 depicts the effective mass and stiffness of one ring spoke.

If one neglects the mass and stiffness of the spokes, the natural frequency of the ring resonator is determined as [16,32]:

(2) $$f_{n}=\frac{1}{2\pi \sqrt{12}}\frac{w_{r}}{{\left(R_{r}\right)}^{2}}\frac{n\left(n^{2}-1\right)}{\sqrt{n^{2}+1}}\sqrt{\frac{E}{\rho }}$$

where $w_{r}$ is the width of the rings and ρ is the mass density. Table 3 depicts the calculated natural frequencies of the ring resonator using Equations (1) and (2) and compares them with FEM.

As seen, the results of Equation (1) and FEM have a 6.8% deviation in the *n* = 3 mode, 6% in the drive mode of *n* = 2, and 37.6% in the sense mode of *n* = 2. The results of Equation (2) show that neglecting the stiffness and mass of spokes yields 19.6% in the *n* = 3 mode, 68.4% in the drive mode of *n* = 2, and 15.1% in the sense mode of *n* = 2. Additionally, it can be concluded that the ring is suitable for the *n* = 3 mode of vibration in which the split frequency is zero.

#### 3.1.2. Ring Resonator Made of Silicon <111> and Spokes Overlapped to the Outside Ring and Anchor

**Figure 3:**
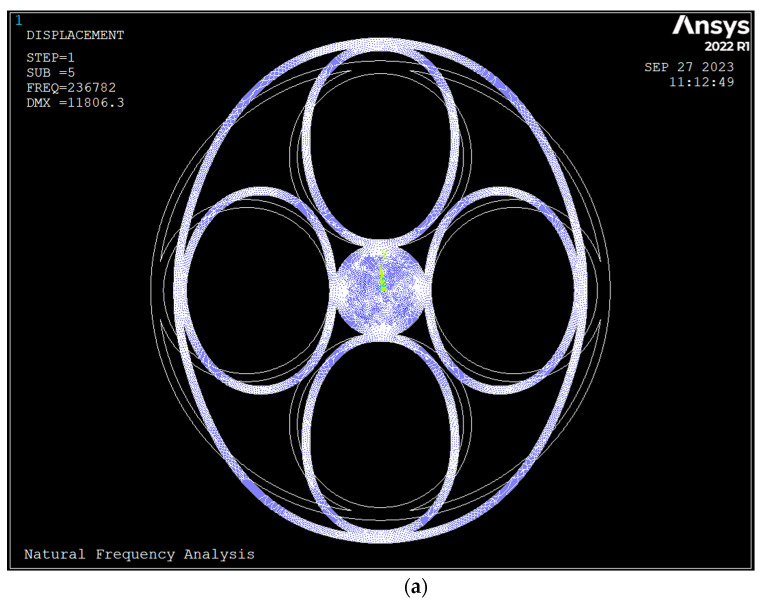

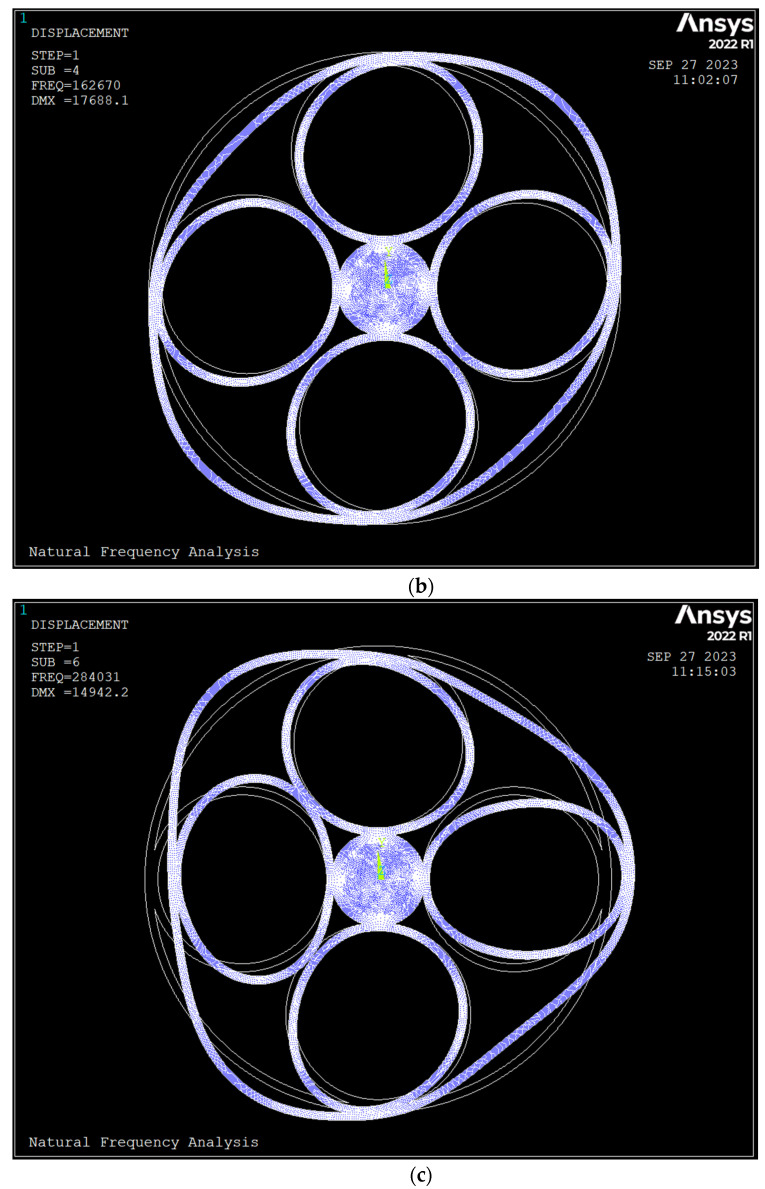

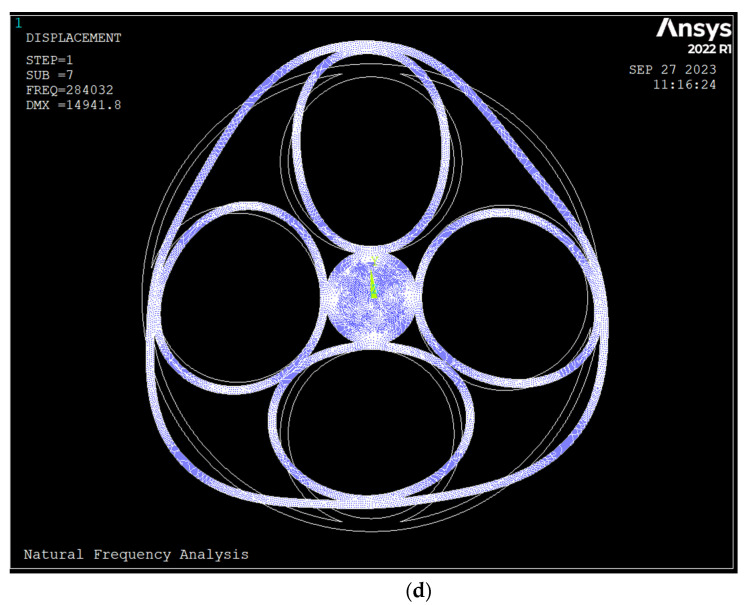
Mode shapes and frequencies of the ring resonator (rings overlapped, silicon <111>); (**a**) *n* = 2 drive mode of vibration *f* = 236.78 kHz; (**b**) *n* = 2 sense mode of vibration *f* = 162.67 kHz; (**c**) *n* = 3 drive mode of vibration *f* = 284.03 kHz; (**d**) *n* = 3 sense mode of vibration *f* = 284.03 kHz.

The ring with a tangent geometry has a weak connection, and in the dynamic analysis, it fails; thus, the radius of the spokes was increased to overlap with the anchor and outside ring. Figure 3 depicts the mode shapes and frequencies of the ring with overlapped geometry. Overlapping of the ring and spokes increases the frequencies of all modes since the stiffness of the ring at the junction point increases without changing the mass. Additionally, the split frequency in the *n* = 2 mode decreased by half, and in *n* = 3, it remained unchanged.

#### 3.1.3. Ring Resonator Made of Silicon <100>, and Spokes Overlapped to the Outside Ring and Anchor

Although isotropic materials have a minimum frequency split, anisotropic silicon is a widely used material for the fabrication of MEMS resonators [33] due to its accuracy and low cost of fabrication. The material properties of silicon depend on orientation relative to the crystal lattice [34]. The orthotropic stiffness matrix for silicon with three axes at [100], [010], and [001] is assumed to be $E_{x}=E_{y}=E_{z}=179\;\mathrm{GPa}$, $v_{xy}=v_{yz}=v_{zx}=0.28$, and $G_{xy}=G_{yz}=G_{zx}=77.5\;\mathrm{GPa}$.

#### 3.1.4. Ring Resonator Made of Silicon <110> and Spokes Overlapped to the Outside Ring and Anchor

The orthotropic stiffness matrix for silicon with three axes at [110], [$\bar{1}$10], and [001] is assumed to be $E_{x}=E_{y}=179\;\mathrm{GPa}$, $E_{z}=130$ $v_{xy}=0.064$, $v_{yz}=0.36$, $v_{zx}=0.28$, and $G_{xy}=50.9\;\mathrm{GPa}$. $G_{yz}=G_{zx}=79.6\;\mathrm{GPa}$.

### 3.2. Pull-in Voltage Analysis

**Figure 4:**
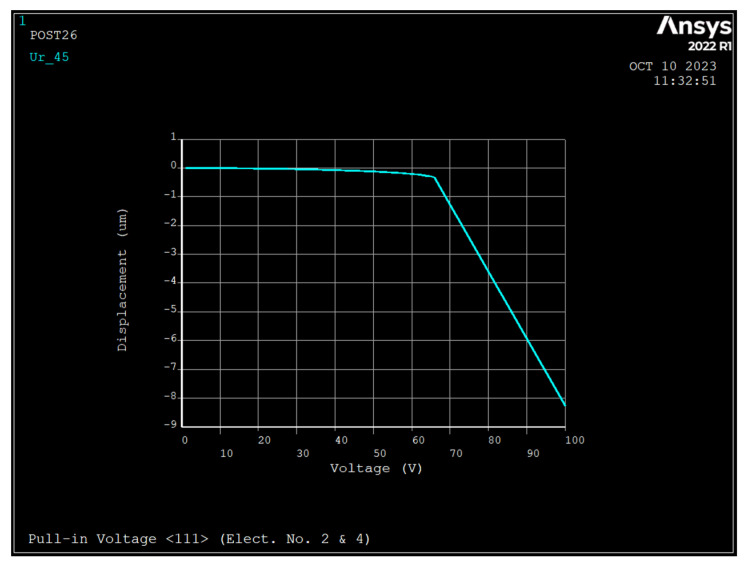
Displacement versus applied DC bias voltage to electrodes number 2 and 4 (1  μm air gap, and silicon <111>). The y axis unit is μm reads um.

**Figure 5:**
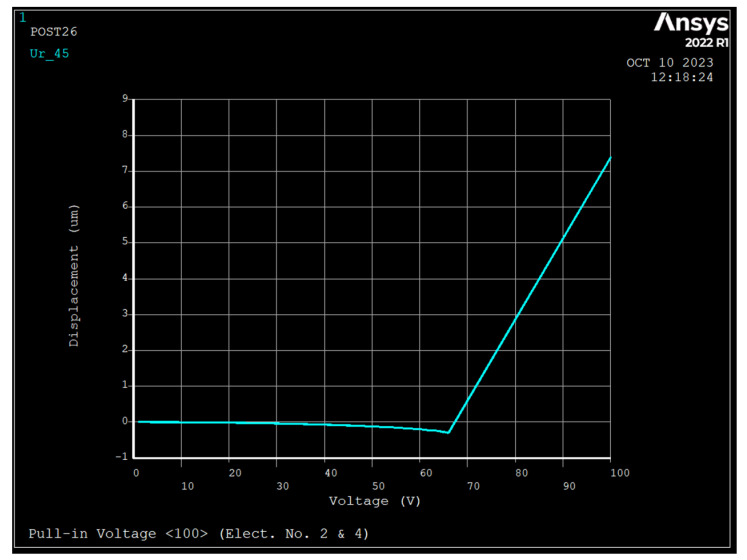
Displacement versus applied DC bias voltage to electrodes number 2 and 4 (1  μm air gap, and silicon <100>).

**Figure 6:**
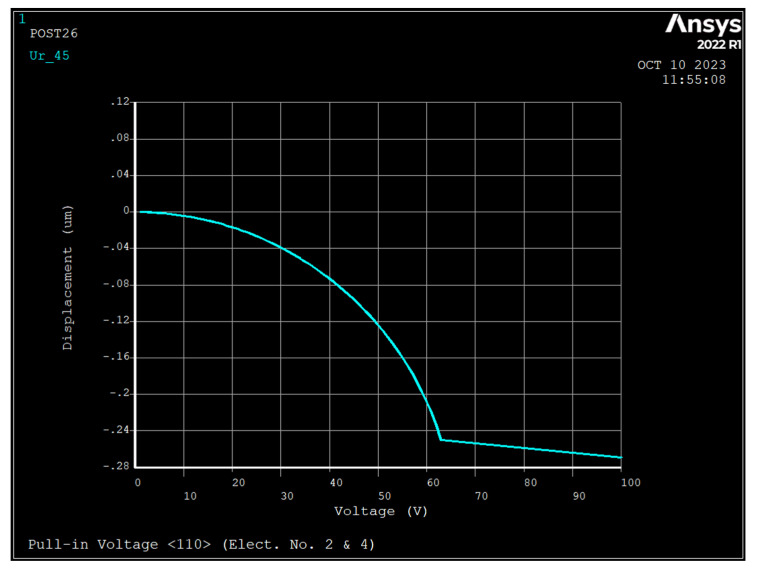
Displacement versus applied DC bias voltage to electrodes number 2 and 4 (1  μm air gap, and silicon <110>).

The pull-in voltage is the maximum DC bias voltage that can be applied to the ring since, for voltages above it, the ring is unable to restore its equilibrium state. A DC bias voltage was applied to electrodes 2 and 4 (Figure 1) and air gap of 1 μm. Figure 4, Figure 5 and Figure 6 depict the radial displacement of the ring at point θ=45 as a function of the applied DC bias voltage.

As seen at DC bias voltages of 67 V, 67 V, and 64 V, the displacement approaches infinity for silicon <111>, <100>, and <110>, respectively, and the pull-in displacements in the radial direction are 0.367 μm, 0.338 μm, and 0.271 μm, respectively, which are almost are one-third of the air gap.

### 3.3. Resonance Frequency Shift

**Figure 7:**
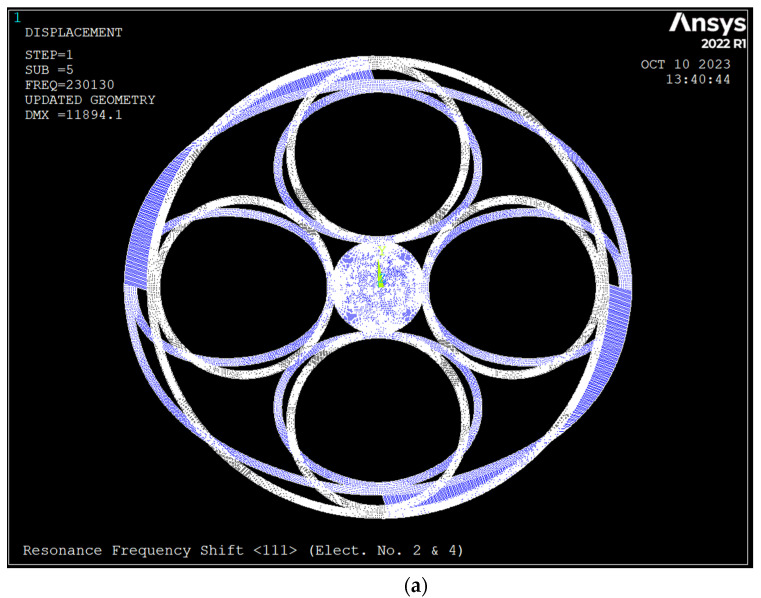

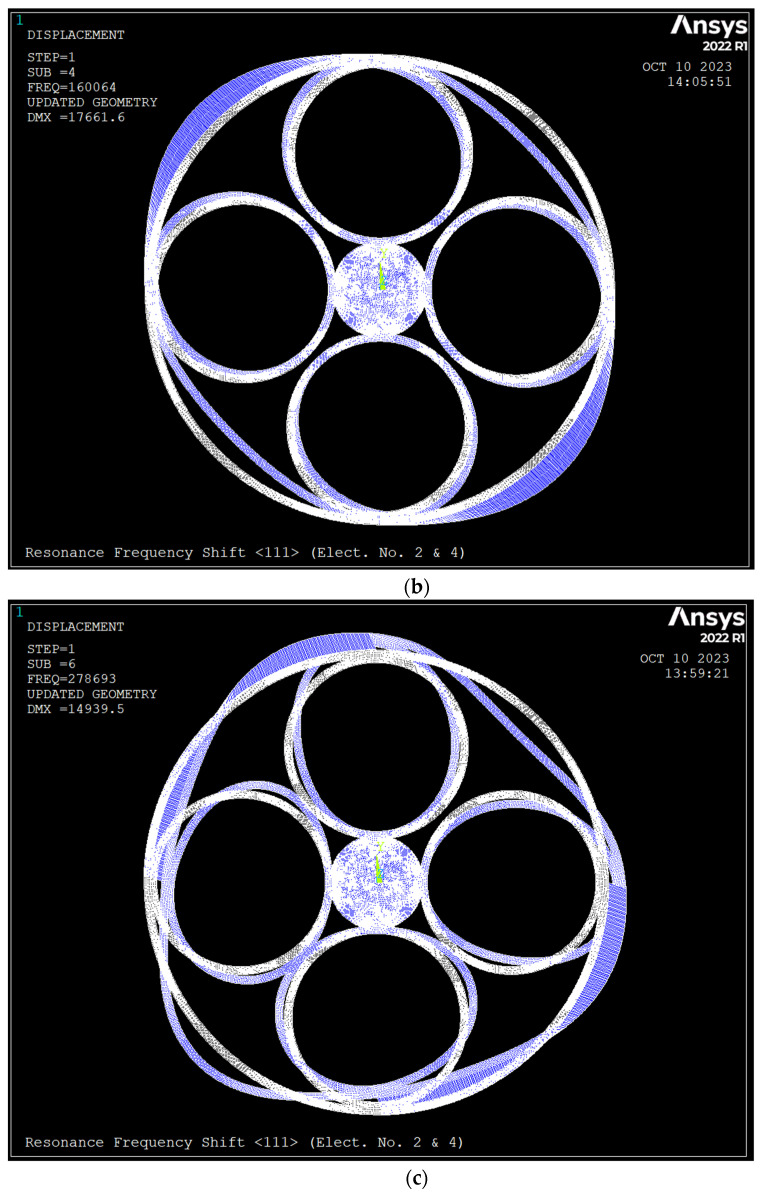

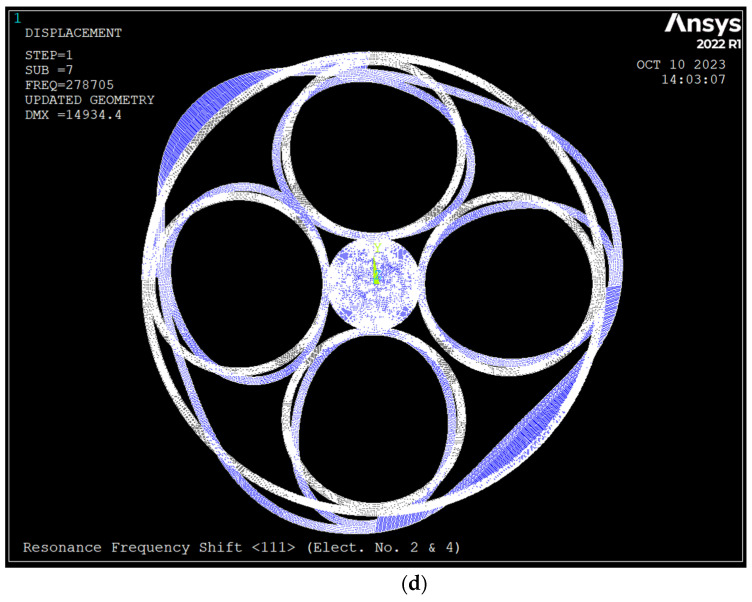
Resonance frequencies shift of the ring resonator due to bias DC voltage of 5 V applied to electrodes number 2 and 4 (silicon <111>); (**a**) Drive mode *n* = 2, frequency 230.13 kHz; (**b**) Sense mode *n* = 2, frequency 160.06 kHz; (**c**) Drive mode *n* = 3, frequency 278.69 kHz; (**d**) Sense mode *n* = 3, frequency 278.71 kHz.

The natural frequency of the ring resonator would shift due to the applied DC bias voltage. Figure 7 depicts the drive and sense mode shapes and frequencies of the ring made of silicon <111> due to a DC bias voltage of 5 V applied to electrodes 2 and 4 and the air gap of 1 μm.

### 3.4. Harmonic Analysis of the Nonrotating Ring

**Figure 8:**
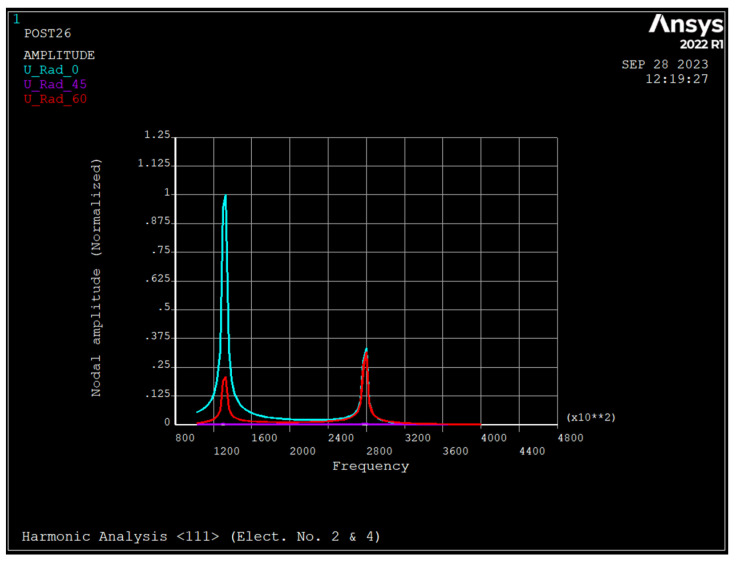
Frequency response of the ring due to applied voltage of 5+0.1 sin wt applied to electrode number 2 and 5−0.1 sin wt to electrode number 4 (silicon <111>).

**Figure 9:**
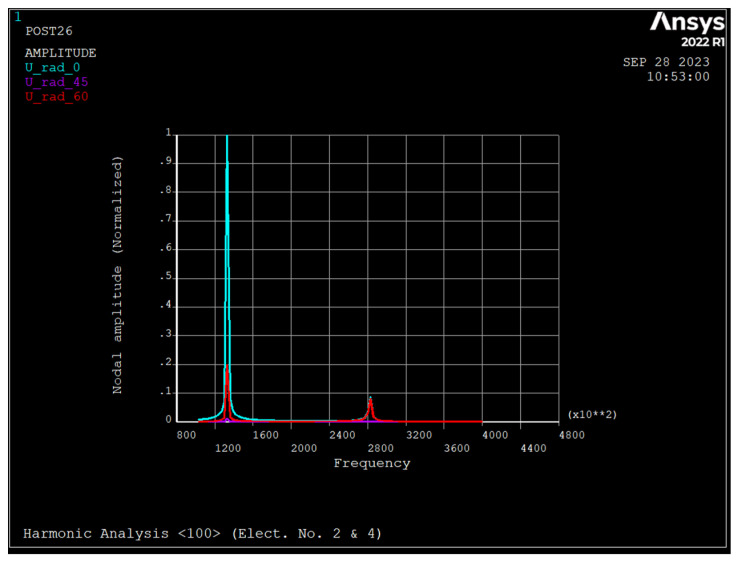
Frequency response of the ring due to applied voltage of 5+0.1 sin wt applied to electrode number 2 and 5−0.1 sin wt to electrode number 4 (silicon <100>).

**Figure 10:**
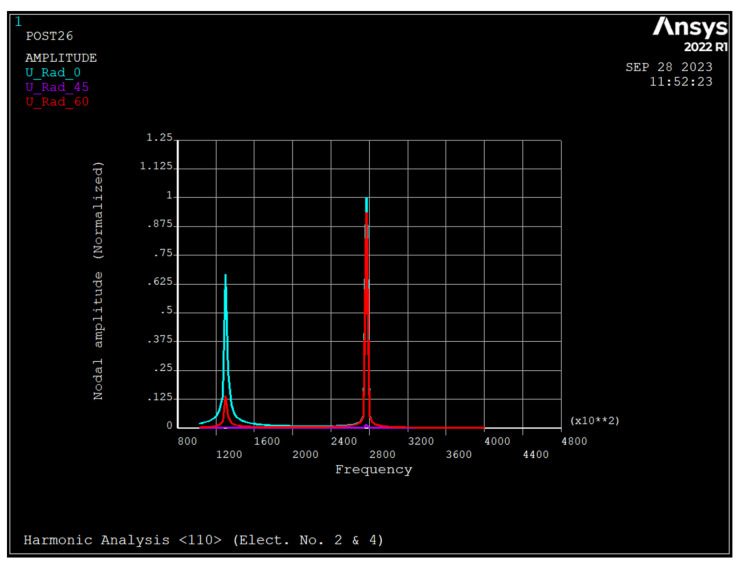
Frequency response of the ring due to applied voltage of 5+0.1 sin wt applied to electrode number 2 and 5−0.1 sin wt to electrode number 4 (silicon <110>).

Figure 8, Figure 9 and Figure 10 depict the frequency response of the ring due to an applied voltage of 5+0.1sinwt (V) to electrode number 2 and 5−0.1sinwt (V) to electrode number 4. As seen, the radial displacement of a point on the ring at θ=45 degrees is zero, which implies that the mode shape is a *n* = 2 sense mode of vibration [31].

As seen, in rings made from silicon <111> and <100>, the major resonance frequency is in the sense mode of *n* = 2, while for rings of <110>, it is in drive mode.

**Figure 11:**
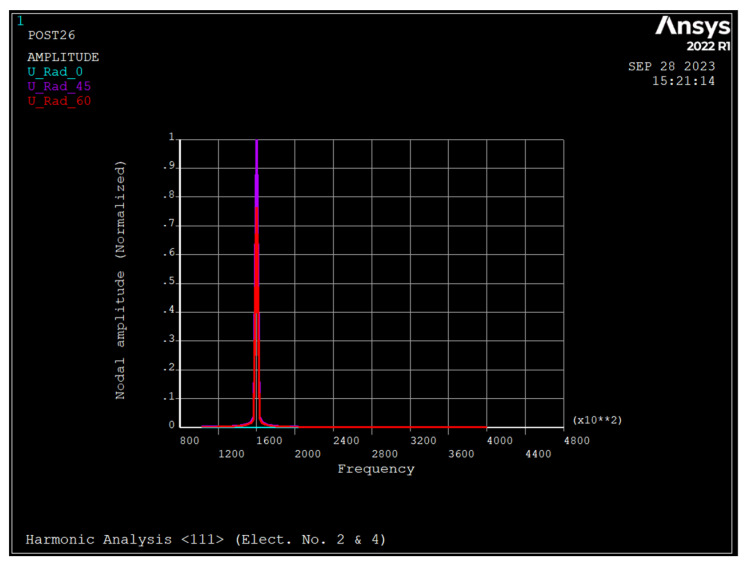
Frequency response of the ring due to applied voltage of 5+0.1 sin wt applied to electrodes number 2 and 4 (silicon <111>).

**Figure 12:**
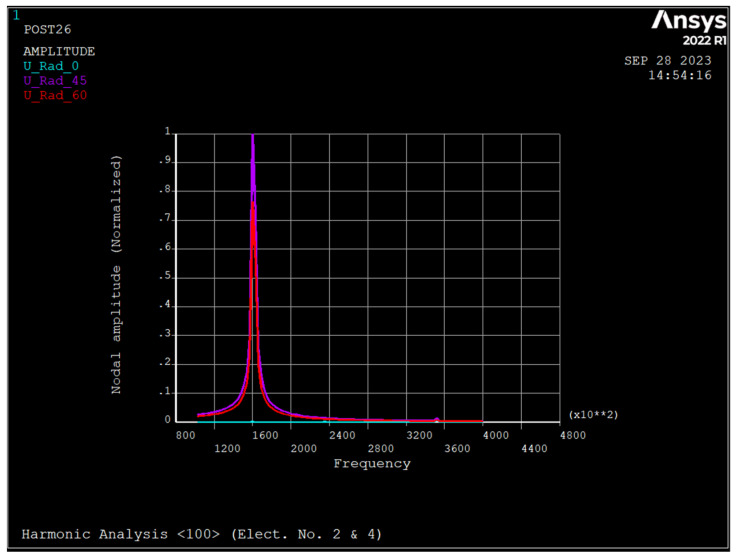
Frequency response of the ring due to applied voltage of 5+0.1 sin wt applied to electrodes number 2 and 4 (silicon <100>).

**Figure 13:**
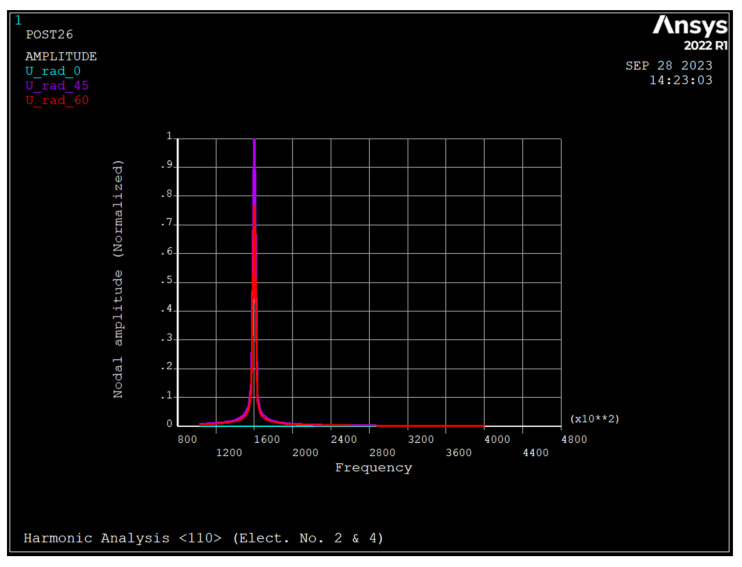
Frequency response of the ring due to applied voltage of 5+0.1 sin wt applied to electrodes number 2 and 4 (Silicon <110>).

Figure 11, Figure 12 and Figure 13 depict the frequency response of the ring due to an applied voltage of 5+0.1sinwt V to electrodes 2 and 4. As seen, the radial displacement of a point on the ring at θ=45 degrees is zero, which implies that the mode shape is *n* = 2 sense mode of vibration [31]. 

Table 4 depict the summary of dynamic analyses of the ring resonator made from different types of silicon.

As seen, silicon <100> has a maximum frequency for both the *n* = 2 and *n* = 3 modes, followed by silicon <111>. For the *n* = 3 mode of vibration, the split frequency for all types of silicon is minimal, and for the *n* = 2 mode, silicon <100> has a maximum frequency split. The pull-in voltage of silicon <110> is the minimum, and silicon <111> and <110> have the same pull-in voltage. The applied DC bias voltage decreases the resonance frequency of all modes regardless of the type of silicon and increases the split frequency of *n* = 3 mode of vibration almost by the same amount. When a voltage of 5+0.1 sin wt (V) is applied to electrodes 2 and 4, the ring resonator has one resonance frequency of 160 kHz, regardless of the type of silicon. However, when 5+0.1 sin wt (V) applies to electrode number 2 and 5−0.1 sin wt (V) applies to electrode number 4, there are two resonance frequencies corresponding to the drive and sense modes in *n* = 2. For silicon <111> and <100>, the primary resonance frequency is the drive mode, while for silicon <110> it is sense mode.

## 4. Device Fabrication and Testing

**Figure 14:**
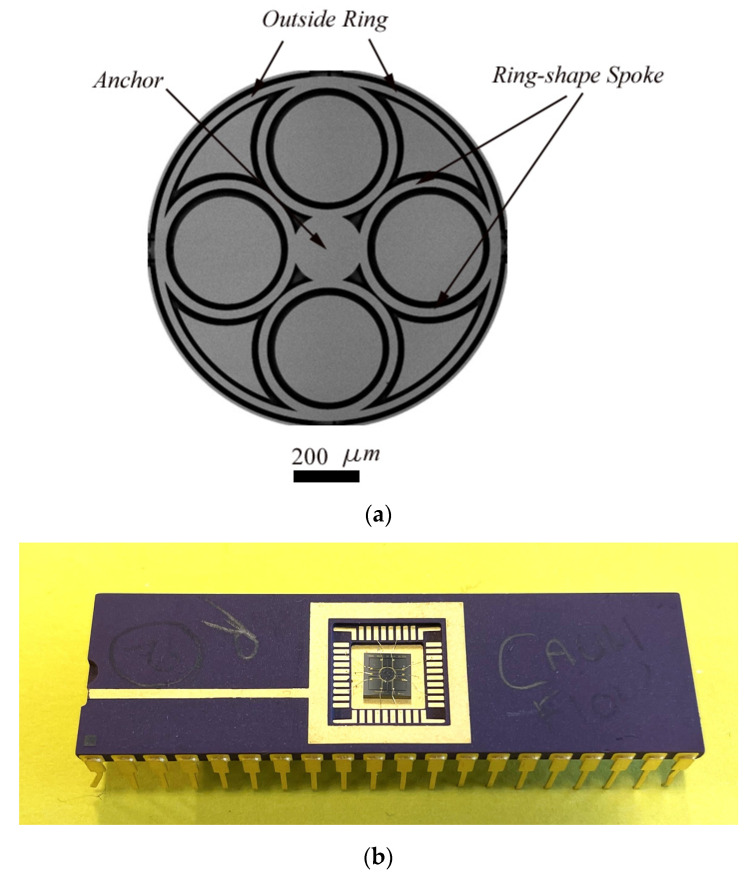
SEM and packaging of fabricated ring resonator with ring-shaped spokes; (**a**) SEM of the fabricated ring resonator showing the ring resonator, electrodes, and electrical connections; (**b**) Prototype packaging using DIP (dual in-line package) Pin 40.

The commercially available silicon on insulator (SOI) MEMS process based on bulk micromachining known as MicraGEM-Si™ [35] was considered for fabrication, as it is a MEMS inertial sensor fabrication process with a suitable device layer thickness modeled in this research. Details of the commercial fabrication process are available in ref. [35]. Although a double SOI-based process is used, the device can also be fabricated using a simple SOI fabrication process with dry etching to define the device layer and electrode followed by wet/vapor timed etching of the oxide layer for device release. Once the fabrication was completed, the total die size was measured as 4 mm × 4 mm. Standard packaging was required to connect the prototype with the measuring devices. The device was packaged in open air for the initial testing. The prototype was wire-bonded to the electrical pad and packaged using a DIP 40 Chip carrier. Figure 14 depicts the SEM and packaging of the fabricated ring resonator based on the chosen dimensions.

**Figure 15:**
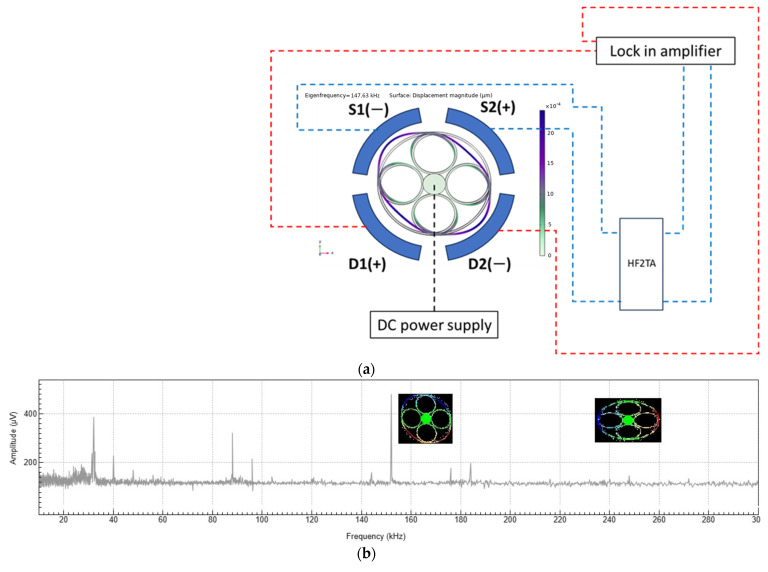
The test arrangement and results; (**a**) The schematic representation of how the device is connected to the lock-in amplifier, where the dotted lines indicate, red for the input electrical signal to the device and blue for the output signal from the device.; (**b**) The frequency sweep response of the ring resonator due to applied voltage to electrodes number 3 and 4 (LIA test).

To measure the resonant frequencies of the ring resonator made from silicon <100>, the lock-in amplifier (HF2LI, Zurich instruments) is used. The device is directly connected to the lock-in amplifier (LIA) through electrodes number 3 and 4 with 180 degrees phase angle, denoted D1 and D2 (Figure 15a), and the response of the device is measured in electrodes number 1 and 2, denoted by S1 and S2 (Figure 15a). The received signal is amplified using a Transimpedance amplifier (HF2TA, Zurich instruments) between the output and lock-in-amplifier. As the frequency carrier signal is generated inside the LIA they are modulated in the device, then this modulated signal is taken inside by getting amplified by HF2TA and demodulated using a reference signal to remove any noise.

Figure 15b shows the frequency sweep plot obtained from the LIA for the ring resonator made from silicon <100>. The sweep was performed from 15 kHz to 300 kHz; as seen, there is a peak around 34 kHz, which corresponds to the torsional mode of vibration as a rigid body. Next, a considerable peak can be seen about 88 kHz, coinciding with the rocking mode of vibration. The third considerable peak at 152 kHz corresponds to the sense mode of *n* = 2, which upon comparison with the results of FEM, 163.82 kHz (Table 2), a deviation of 7.7% is observed. The last peak corresponds to a frequency of 248 kHz, corresponding to the drive mode of *n* = 2; compared with the results of FEM, 241.37 kHz (Table 2), a deviation of 2.2% is observed. It is worth mentioning that due to the configuration of the applied voltage, the amplitude of the sense mode is higher than the drive mode.

## 5. Conclusions

A MEMS resonator based on a novel ring resonator was designed, analyzed, and fabricated, and its dynamic behavior was tested. The frequency and mode shapes of the ring resonator were analyzed analytically and numerically. The results show that the ring resonator has good performance in mode *n* = 3 with a very low-frequency split. Pull-in voltage, resonance shift frequency, and harmonic analyses are carried out. The prototype was tested to capture its resonance frequency. The results show that the experimental resonance frequency is 300.56 kHz, which is within a 4.4% deviation from the FEA results. The proposed novel resonator design is simple, requiring only one-step alignment between the anchor and the device layer. It is suitable for SOI-based fabrication, and it involves inner ring-shaped suspension springs and one ring-shaped mass. Additionally, the novel inner suspension design creates many research opportunities in terms of investigating the number of rings, arrangement of electrodes, number of electrodes, transduction methods, energy loss mechanisms and vacuum packaging. There are also opportunities for future work, including prototype fabrication using a single SOI wafer in a clean room, testing under a vacuum to characterize the Q factor, and dynamics and vibration testing for detailed characterization. Such a simple resonator and gyro design can further aid the development of mode-matched gyroscopes for sensing velocity and orientation in consumer, automotive, navigation and robotic applications.

## Figures and Tables

**Table 1:** Initial values of the considered design parameters of the proposed resonator.

Design Parameters	Values (μm)
Anchor radius, $R_{A}$	120
Outside ring radius (mean), $R_{r}$	583
Height of the ring, $H_{r}$	30
Width of the rings, $W_{r}$	20
Height of the spoke, $H_{s}$	30
Width of the spoke, $\;W_{s}$	20.5
Air gap, *d*	10

**Table 2:** Effective mass and stiffness of one ring spoke.

Effective Mass (kg)		Stiffness (N/m)	
Radial Direction	Tangential Direction	Radial Direction	Tangential Direction
$8.62\times 10^{-10}$	$8.86\times 10^{-10}$	2230	422.43

**Table 3:** Frequency of the ring resonator (kHz) shown in Figure 1 calculated using different methods.

Method	*n* = 2, Mode		*n* = 3, Mode	
	Drive Mode	Sense Mode	Drive Mode	Sense Mode
FEM	201.59	55.27	223.82	223.82
Equation (1)	189.59	76.07	208.58	208.58
Equation (2)	63.60	63.60	179.88	179.88

**Table 4:** Summary of numerical analysis of ring resonators with different types of silicon.

Dynamic Behavior			Type of Silicon		
			<111>	<100>	<110>
Natural Frequency Analysis	*n* = 2, mode (kHz)	Drive	236.78	241.37	234.78
		Sense	162.67	163.82	162.16
	*n* = 3, mode (kHz)	Drive	284.03	287.72	282.47
		Sense	284.03	287.72	282.47
Pull-in Voltage (air gap 1 μm)	Voltage (V)		67	67	64
	Displacement (μm)		0.367	0.338	0.271
$\mathrm{Resonance}\;\mathrm{Frequency}\;\mathrm{Shift}\;\mathrm{Analysis}\;(\;V_{dc}=5$ V, air gap 1 μm)	*n* = 2, mode (kHz)	Drive	230.13	228.18	234.24
		Sense	160.06	159.56	162.22
	*n* = 3, mode (kHz)	Drive	278.69	277.19	282.24
		Sense	278.71	277.20	282.25
Harmonic Analysis (Resonance frequency, kHz)	5±0.1 sin wt (V)	Primary mode	280 (*n* = 3)	283 (*n* = 3)	130 (*n* = 2)
		Secondary mode	133 (*n* = 2)	133 (*n* = 2)	277 (*n* = 3)
	5+0.1 sin wt (V)		160 (*n* = 3)	160 (*n* = 3)	160 (*n* = 3)

## Data Availability

The datasets used and/or analyzed during the current study are available from the corresponding author upon reasonable request.
